# Adaptive immune responses are altered in adult mice following neonatal hyperoxia

**DOI:** 10.14814/phy2.13577

**Published:** 2018-01-25

**Authors:** Vasantha H. S. Kumar, Huamei Wang, Lori Nielsen

**Affiliations:** ^1^ Department of Pediatrics University at Buffalo Buffalo New York

**Keywords:** Adaptive immunity, BPD, gene expression, genes, hyperoxia, p21, T cells

## Abstract

Premature infants with bronchopulmonary dysplasia (BPD), are at risk for frequent respiratory infections and reduced pulmonary function. We studied whether neonatal hyperoxia disrupts adaptive immune responses in adult mice, contributing to higher respiratory‐related morbidities seen in these infants. Newborn mice litters were randomized at 3 days to 85% O_2_ or room air (RA) for 12 days. Whole lung mRNA was isolated in both the groups at 2 weeks and 3 months. Gene expression for T‐cell and B‐cell adaptive immune response was performed by real‐time PCR and qRT‐PCR; protein expression (p21, IL4, IL10, IL27, cd4) was performed by enzyme immunoassay along with p21 immunohistochemistry. Hyperoxia increased expression of p21 and decreased expression of 19 genes representing T/B‐cell activation by ≥ fourfold; three of them significantly (Rag1, Cd1d1, Cd28) compared to the RA group at 2 weeks. Despite RA recovery, the expression of IFN *γ*, IL27, and CD40 was significantly reduced at 3 months in the hyperoxia group. Expression of p21 was significantly higher and IL27 protein lower at 2 weeks following hyperoxia. Adult mice exposed to neonatal hyperoxia had lower IL4 and IL10 in the lung at 3 months. Adaptive immune responses are developmentally regulated and neonatal hyperoxia suppresses expression of genes involved in T‐/B‐cell activation with continued alterations in gene expression at 3 months. Dysfunction of adaptive immune responses increases the risk for susceptibility to infection in premature infants.

## Introduction

Bronchopulmonary dysplasia is the most common form of chronic lung disease following premature birth, especially in extremely low‐birth‐weight (ELBW) infants. Several prenatal (Morrow et al. 2017), postnatal (Jobe 2011), and genetic factors (Bhandari and Gruen 2006) act on the immature lung leading to development of BPD. Multifactorial origin of the disease makes it especially difficult to treat these infants, with no specific therapies in sight. Despite recent advances such as gentler ventilation strategies and surfactant, the incidence of BPD has not changed over the last few decades, reflecting improved survival of extremely low‐birth‐weight infants who are at highest risk for BPD (Schmidt et al. 2015).

Multiple factors are implicated in the pathogenesis of BPD, including chorioamnionitis (Eriksson et al. 2014), sepsis (Shah et al. 2015), mechanical ventilation (Allison et al. 2008), and supplemental oxygen use (Jobe and Kallapur 2010) in these infants. Resulting pulmonary inflammation leads to recruitment of neutrophils and monocytes in the lungs, production of pro‐inflammatory cytokines, and development of adaptive immunity for resolution of infection and inflammation. However, persistent inflammation, resulting from environmental factors such as mechanical ventilation or hyperoxia in a setting of an immature lung, is a characteristic feature of BPD (Balany and Bhandari 2015). Overall cytokine pattern in infants who developed BPD or died indicated an impairment in transition from innate immune response mediated by neutrophils to adaptive immune response mediated by T lymphocytes (Ambalavanan et al. 2009). Adaptive immune responses are subordinate to innate immune responses, in that, mononuclear phagocytic cells and antigen presenting cells are required for effective lymphoid responses to antigens (Hoebe et al. 2004). The gradual development of immunity during gestation is worsened not only by premature birth but also by the immaturity of the cellular immune response at birth (Gasparoni et al. 2003).

Adult mice exposed to neonatal hyperoxia demonstrate enhanced inflammation, suggesting that neonatal hyperoxia not only disrupts lung development but also reprograms key innate immunoregulatory molecules in the lung contributing to viral disease (O'Reilly et al. 2008). Neonatal hyperoxia may alter the host response to respiratory viral infection in adult mice by inducing long‐term changes in the reparative or the cytotoxic nature of natural killer cells (Reilly et al. 2015). We have shown that the absolute lymphocyte count in lung lavage is higher, with an increase in CD3 cell count in the lung in adult mice following neonatal hyperoxia (Kumar et al. 2016). Extremely low‐birth‐weight infants, who are most risk for BPD, are also at increased risk for dysfunctional innate and adaptive immune responses in the postnatal period. We hypothesize that adaptive immune responses are developmentally regulated and hence its disruption by factors such as hyperoxia may lead to dysfunctional immune response. We studied the T‐cell and B‐cell immune responses in adult mice in a neonatal hyperoxia model of lung injury similar to BPD in infants.

## Methods

### Oxygen exposure

This is an in vivo study of hyperoxic lung injury in newborn mice to mimic to the pathophysiology of BPD in premature infants. The study was approved by IACUC of the University at Buffalo. Time dated pregnant C57/BL6 mice were allowed to acclimate in the animal facility a week prior to delivery. On postnatal day 3, newborn mice litters were randomized to receive either 85% O_2_ (hyperoxia) or room air (RA) for 12 days (P3–P15; humidity: 50–60%). Room air mice were subjected to the identical environment as hyperoxia‐exposed mice. Dams were alternated between RA and oxygen exposed litters every 24 h to prevent maternal O_2_ toxicity. Mice were recovered in room air at the end of hyperoxia exposure at P15 in both the groups. Mice were killed at the end of study (P15) and at 3 months by intraperitoneal injection of sodium pentobarbital. Lung tissues was collected for gene expression studies and for formalin fixation at 2 weeks and 3 months of age (*N* = 6 in each group, each time point).

### RNA isolation

Lung tissue was flash frozen in liquid nitrogen and stored at −80°C until processed. RNA was isolated from flash frozen mouse lung using RNeasy Mini kit (Qiagen, Valencia, CA) with on column DNase digestion per manufacturer's protocol. RNA integrity was assessed using Experion Automated Electrophoresis System (Bio‐Rad, Hercules, CA).

### Whole lung gene expression profiling by RT^2^ qPCR

Mouse T‐cell and B‐cell activation RT^2^ PCR array (SA Biosciences, MD) profiles the expression of 84 genes representing T‐cell and B‐cell activation, a key part of adaptive immunity. The array includes genes involved in T‐cell and B‐cell activation, proliferation, and differentiation. Using RT^2^ first strand kit (SA Biosciences, MD), 300 ng of total RNA was reverse transcribed to cDNA, which was mixed with RT^2^‐SYBR Green qPCR master mix. Aliquots of this mix was placed into each of the PCR array plates containing the predispensed gene‐specific primer sets and PCR performed on a 96‐well MyiQ thermocycler (Bio‐Rad, Herculus, CA) according to manufacturer's protocol. The instrument's software was used to calculate the threshold cycle (C_t_) values for all the genes on the PCR Array. Fold change in gene expression for pair‐wise comparison was processed using the excel‐based PCR Array Data Analysis software (SA Biosciences) by 2^−∆∆C (*t*)^ method, comparing to the corresponding RA group (RA 2 weeks; RA 3 months).

### Quantitative reverse transcription PCR (RT‐qPCR)

Genes with significant expression (Cd1d1, Il27, Cd28, Cd4 and p21) from the gene expression profiling were subjected to RT‐qPCR. Total cellular RNA was reverse transcribed using iScript cDNA Synthesis kit (Bio‐Rad, Hercules, CA). Reactions containing no reverse transcriptase were included for individual RNA. Primers were purchased from Real Time Primers (Elkins Park, PA), and reference genes *β*‐actin, Rpl13A and Pgk1 (Genorm Software, Biogazelle, Belgium) were used per protocol. PCR was run in duplicates in all samples in a CFX Connect Real‐Time PCR machine (Bio‐Rad) using SYBR Green. Results for these primers were analyzed using the online data analysis software (SA Biosciences, MD).

### Protein analysis

Quantitative determination of interleukin‐4 (Il4), interleukin‐10 (Il10), interleukin‐27 (1 l27), cd4 antigen (cd4) and cyclin‐dependent kinase inhibitor 1A (cdkn1a or p21) was performed in cell‐free supernatants of lung homogenates in both the oxygen and the room air groups in 2 weeks and 3 months of age. Quantikine mouse Il4 and Il10 immunoassay (R & D Systems, Minneapolis, MN), p21 EIA (Abcam, Cambridge, MA), Il27 enzyme immunoassay (Boster Biologicals, Pleasanton, CA), and cd4 ELISA kit (Xpress Bio, Frederick, MD) were used to determine whether the levels of protein in lung tissue extracts are per manufacturer's protocol. All experimental groups were tested in duplicates. The cytokine levels were normalized to the protein present in cell‐free preparation of each sample measured by Lowry assay and expressed as either pg/mg or ng/mg lung protein.

### p21 Immunohistochemistry

Immunostaining was performed on lung sections for the cell cycle inhibitor, p21. Antigen was first retrieved in paraffin‐embedded sections by heating in pH 6.0 citrate buffer (Lab‐Vision, Fremont, CA) for 20 min. Slides were washed in PBS and incubated with 2% BSA to block nonspecific binding. Lung sections were incubated for 60 min at 4°C with mouse monoclonal antibody to p21 (1:100 dilution; Santa Cruz # 6246) and rinsed with PBS. The sections were then incubated with peroxidase labeled rabbit anti‐mouse IgG (1;1000) for 30 min at 37°C, washed with PBS, and stained with DAB staining kit (Dako Envision_HRP‐DAB; Carpinteria, CA). Nonspecific IgG and omission of primary antibody were used as controls for staining specificity. Quantification of p21 staining was done at 400× and the number of p21 stained nuclei per high‐power field (56,000 *μ*m^2^) of lung cross‐sectional area was determined. Twelve random HPFs per animal (*n* = 5 in each group at 2 weeks/3 months) were evaluated. The identity of the sample was masked to the observer estimating IHC staining to avoid bias. The score was given according to the intensity of the nuclear or cytoplasmic staining (no staining = 0; weak staining = 1; moderate staining = 2; strong staining = 3) and the extent of stained cells (0% = 0; 1–10% = 1; 11–50% = 2; 51–80% = 3; 81–100% = 4). The final immunoreactive score was determined by multiplying the intensity and the extent of positivity scores of stained cells, with the minimum score of 0 and maximum score of 12 (Han et al. 2009).

### Statistical analysis

All data were expressed as mean ± standard deviation (SD) with *n* representing the number of animals studied (*N* = 6 in each group). *P* values are calculated based on Students' *t*‐test of the replicate 2^−∆∆C(*t*)^ values for each gene in the control group and the treatment group. A *P* value of <0.05 was considered significant. Differences among groups were compared by ANOVA with Bonferroni–Dunn post hoc test when appropriate.

## Results

### Hyperoxia suppresses genes involved in T‐cell and B‐cell activation at 2 weeks

Of the 84 genes in the PCR array, there was widespread suppression of gene expression involved in B‐cell and T‐cell function following 12 days of hyperoxia (Table 1). Forty‐two percent (35/84) of the genes were underexpressed by ≥2‐fold; 22% (19/84) genes were underexpressed by ≥4‐fold (Table 1). The genes involved in ≥4‐fold underexpression compared to the room air group were involved in regulation, activation, proliferation, differentiation of T or B cells (Table 1). Genes that were significantly downregulated included interleukin 4 (Il4), recombination activation gene 1(Rag1), cd1d1, and cd28. Suppression of immune regulation genes was accompanied by significant overexpression of cyclin‐dependent kinase inhibitor 1A (p21), a cell cycle inhibitor (Table 1). All but one gene (p21) were underexpressed to a greater or lesser degree compared with the room air group.

**Table 1:** Real‐time PCR array analysis of T‐cell and B‐cell genes in lung homogenate of mice exposed to 85% O_2_ for 12 days from P3 to P15

Gene Symbol	Gene Description	Fold Change of 85% O_2_ – 2‐week group (compared with 21% O_2_ – 2 weeks)
Genes involved in B‐cell activation		
Cr2	Complement receptor 2	−4.73
Igbp1b	Immunoglobulin (CD79A) binding protein 1b	−12.46
B‐cell differentiation		
Il10	Interleukin 10	−6.06
Il4	Interleukin 4	−5.31*
Nkx2‐3	NK2 transcription factor related, locus 3 (Drosophila)	−5.32
Rag1	Recombination activating gene 1	−4.16*
Regulators of T‐cell activation		
Cd1d1	CD1d1 antigen	−13.05*
Cd3d	CD3 antigen, delta polypeptide	−4.20
Cd8a	CD8 antigen, alpha chain	−5.43
Irf4	Interferon regulatory factor 4	−4.86
Prlr	Prolactin receptor	−22.70
T‐cell proliferation		
Cd3e	CD3 antigen, epsilon polypeptide	−5.51
T‐cell differentiation		
Ap3b1	Adaptor‐related protein complex 3, beta 1 subunit	−12.68
Cd4	Cd4 antigen	−39.71
Il27	Interleukin 27	−6.75
Th1/Th2 differentiation		
Cd28	CD28 antigen	−7.06*
Cd40lg	CD40 ligand	−17.30
Other genes related to immune cell activation		
H60a	Histocompatibility 60a	−14.90
Overexpressed gene		
Cdkn1a	Cyclin‐dependent kinase inhibitor 1A (P21)	7.70*

Genes in the 85% O_2_ 2‐week group were expressed as the fold change of expression of the same gene in the RA 2‐week group (control group; gene expression in the control group = 1.0). Genes that are under (≤4.0) or over (≥4.0) expressed (relative to RA 2‐week group or the control group) or significant are shown (*n* = six in each group); *P < 0.05 versus RA 2 weeks by Student's *t*‐test. A negative sign prefixes underexpressed genes. Housekeeping genes – Gapdh and Hprt; some genes act at more than one phase of B‐/T‐cell activation.

### Immune gene expression recovers in room air following hyperoxia by 3 months

Following room air recovery up to 3 months, expression of immune‐related genes recovered for the most part. Thirty‐three percent (24/84) of the genes were overexpressed by ≥2‐fold; 14% (12/84) genes were overexpressed by ≥4‐fold (Table 2). Most of the genes that were overexpressed at 3 months (cd1d1, cd3d, cd3e, cd4, cd28, cd40Ig, Ap3b1) were underexpressed at 2 weeks following hyperoxia (Table 2). Genes that were significantly overexpressed at 3 months included cd3d, cd3e, il27, cd28, cd40 ligand, tumor necrosis factor (ligand) superfamily‐13b (Tnfsf13b), and interferon gamma (IFN*γ*) (Table 2). The cell cycle inhibitor, p21, was significantly underexpressed at 3 months (Table 2). Only two other genes to be underexpressed included early growth response 1 (Egr1; FR: −3.8) and recombination activating gene 1(Rag1; FR: −4.81, Table 2).

**Table 2:** Real‐time PCR array analysis of T‐cell and B‐cell genes in lung homogenate of mice exposed to 85% O_2_ for 12 days from P3 to P15

Gene symbol	Gene description	Fold change of 85% O_2_ – 3‐month group (compared with 85% O_2_ – 2 weeks)
Overexpressed genes at 3 months		
Clcf1	Cardiotrophin‐like cytokine factor 1	4.78
Cd1d1	CD1d1 antigen	4.24
Cd3d	CD3 antigen, delta polypeptide	4.27*
Sit1	Suppression inducing transmembrane adaptor 1	4.12
Cd3e	CD3 antigen, epsilon polypeptide	7.96*
Ap3b1	Adaptor‐related protein complex 3, beta 1 subunit	5.45
Cd4	Cd4 antigen	6.59
Il27	Interleukin 27	4.67*
Cd28	CD28 antigen	5.15*
Cd40lg	CD40 ligand	5.37*
Tnfsf13b	Tumor necrosis factor (ligand) superfamily‐13b	5.10*
Ifn*γ*	Interferon gamma	7.10*
Underexpressed genes at 3 months		
Cdkn1a	Cyclin‐dependent kinase inhibitor 1A (P21)	−4.29*
Rag1	Recombination activating gene 1	−4.81

Genes in the 85% O_2_ 3‐month group were expressed as a fold change of expression of the same gene in the 85% O_2_ 2‐week group (control group; gene expression in the control group = 1.0). Genes that are under (≤4.0) or over (≥4.0) expressed (relative to 85% O_2_ 2 weeks or the control group) or significant are shown (*n* = six in each group); *P < 0.05 versus RA–2 weeks by Student's *t*‐test. A negative sign prefixes underexpressed genes. Housekeeping genes – Gapdh and Hprt.

### Developmental regulation of immune gene expression

Among the immune genes studied, several genes were downregulated by ≥5‐fold at 3 months compared with 2 weeks in the room air group (Table 3). Genes with significant downregulation of expression in the lung included Cd1d1 antigen and recombination activating gene 1 (Rag1). This is in contrast to overexpression of genes in the hyperoxia group at 3 months (Table 2). This suggests that immune gene expression is relatively greater early in development and tapers off in adult mice.

**Table 3:** Real‐time PCR array analysis of T‐cell and B‐cell genes in lung homogenate of mice exposed to room air

Gene symbol	Gene description	Fold change in 21% O_2_ – 3‐month group (compared with 21% O_2_ – 2 weeks)
Overexpressed genes at 3 months		
Tnfsf13b	Tumor necrosis factor (ligand) superfamily – 13b	5.25*
Underexpressed genes at 3 months		
Cd1d1	CD1d1 antigen	−6.26*
Cd4	Cd4 antigen	−6.59
H60a	Histocompatibility 60a	−8.83
Hells	Helicase, lymphoid specific	−5.67
Igbp1b	Immunoglobulin‐binding protein 1b	−7.38
Il10	Interleukin 10	−10.0
Prlr	Prolactin receptor	−12.48
Rag1	Recombination activating gene 1	−30.30*

Genes in the 21% O_2_ 3‐month group were expressed as the fold change of expression of the same gene in the 21% O_2_ 2‐week group (control group; gene expression in the control group = 1.0). Genes that are under (≤4.0) or over (≥4.0) expressed (relative to 21% O_2_ 2 weeks or the control group) or significant are shown (*n* = six in each group); *P < 0.05 versus RA–2 weeks by Student's *t*‐test. (A negative sign prefixes underexpressed genes). Housekeeping genes – Gapdh and Hprt.

### Hyperoxia alters immune gene expression in adult mice

Despite prolonged recovery in room air, several of the immune regulation genes in the lung were downregulated in the hyperoxia group at 3 months (Table 4). Interferon gamma (IFN*γ*)), interleukin 27 (Il27), and CD 40 antigen (cd40) were significantly downregulated in the hyperoxia group compared with the room air group at 3 months (Table 4). Secreted phosphoprotein 1 (Spp1) and early growth response 1 (Egr1) gene were downregulated by ≥2‐fold compared with the room air group.

**Table 4:** Real‐time PCR array analysis of T‐cell and B‐cell genes in lung homogenate of mice exposed to 85% O_2_ for 12 days (P3 to P15)

Gene symbol	Gene description	Fold change of 85% O_2_ – 3‐month group (compared with 21% O_2_ – 3 months)
Genes involved in B‐cell activation		
Spp1	Secreted phosphoprotein 1	−3.7
Ifng	Interferon gamma	−2.7*
Egr1	Early growth response 1	−2.4
IL27	Interleukin 27	−2.7*
CD40	CD 40 antigen	−2.0*

Genes in the 85% O_2_ 3‐month group were expressed as the fold change of expression of the same gene in the RA 3‐month group (control group; gene expression in the control group = 1.0). Genes that were under (≤2.0) or over (≥2.0) expressed (relative to control: RA 3‐month group) or significant are shown (*n* = six in each group); *P < 0.05 versus RA 3 months by Student's *t*‐test (a negative sign prefixes underexpressed genes). Housekeeping genes – Gapdh and Hprt; some genes act at more than one phase of B‐/T‐cell activation.

### RT‐qPCR analysis of selected immune genes

The analysis of selected genes was to confirm the trend we noticed in real‐time PCR. CD1D1, CD28, and IL27 were all underexpressed and p21 overexpressed in the hyperoxia group at 2 weeks compared with room air group (CD1D1: −6.05; CD28: −2.55; IL27: −3.85; p21: +19.80). The same genes were overexpressed except p21 in the hyperoxia groups at 3 months (CD1D1: 2.05; CD28: 3.72; IL27: 2.48; p21: −6.64 vs. 85% O_2_ 2 weeks). Interleukin‐27 was underexpressed in the hyperoxia group compared with room air group at 3 months (IL‐27: −2.18). The trend in expression was similar in both real‐time PCR and qRT‐PCR for these selected genes.

### Immune protein expression

**Figure 1:**
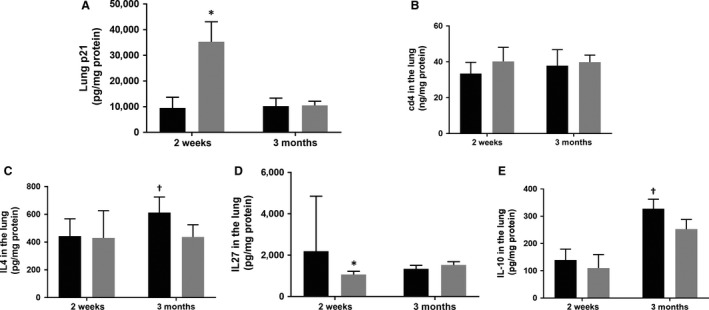
Protein expression of selected molecules in whole lung homogenate in room air and the hyperoxia (85% O_2_) groups (columns with black fill – room air groups; columns with gray fill – hyperoxia groups). Cell cycle inhibitor, p21 (1A), cluster of differentiation 4 (CD4) glycoprotein (1B), immune‐regulatory cytokines interleukin‐4 (IL4) (1C), and interleukine‐27 (IL27) (1D); and inteleukin‐10 (IL10) (1E), an anti‐inflammatory cytokine was measured in the lung by enzyme immunoassay (see text for details). *P < 0.05 versus room air – 2 weeks; †*P* < 0.05 versus hyperoxia – 3 months, ANOVA. *N* = six in each group, each time point.

Whole lung p21 expression was significantly higher following hyperoxia at 2 weeks (*P < 0.05 vs. room air – 2 weeks, Fig. 1A); however, there was no difference in p21 expression at 3 months between the groups (Fig. 1A). Expression of interleukin‐4 (Fig. 1C) and interleukin‐10 (Fig. 1E) was significantly higher in the room air group at 3 months compared with hyperoxia group (^†^ *P* < 0.05 vs. hyperoxia – 3 months). Expression of interleukin‐27 was significantly higher in the room air group at 2 weeks (*P < 0.05 vs. room air – 2 weeks, Fig. 1D); however, there were no differences at 3 months between the groups (Fig. 1D). Expression of cd4 protein was not different between the groups at 2 weeks or at 3 months (Fig. 1B).

### p21 immunohistochemistry

**Figure 2:**
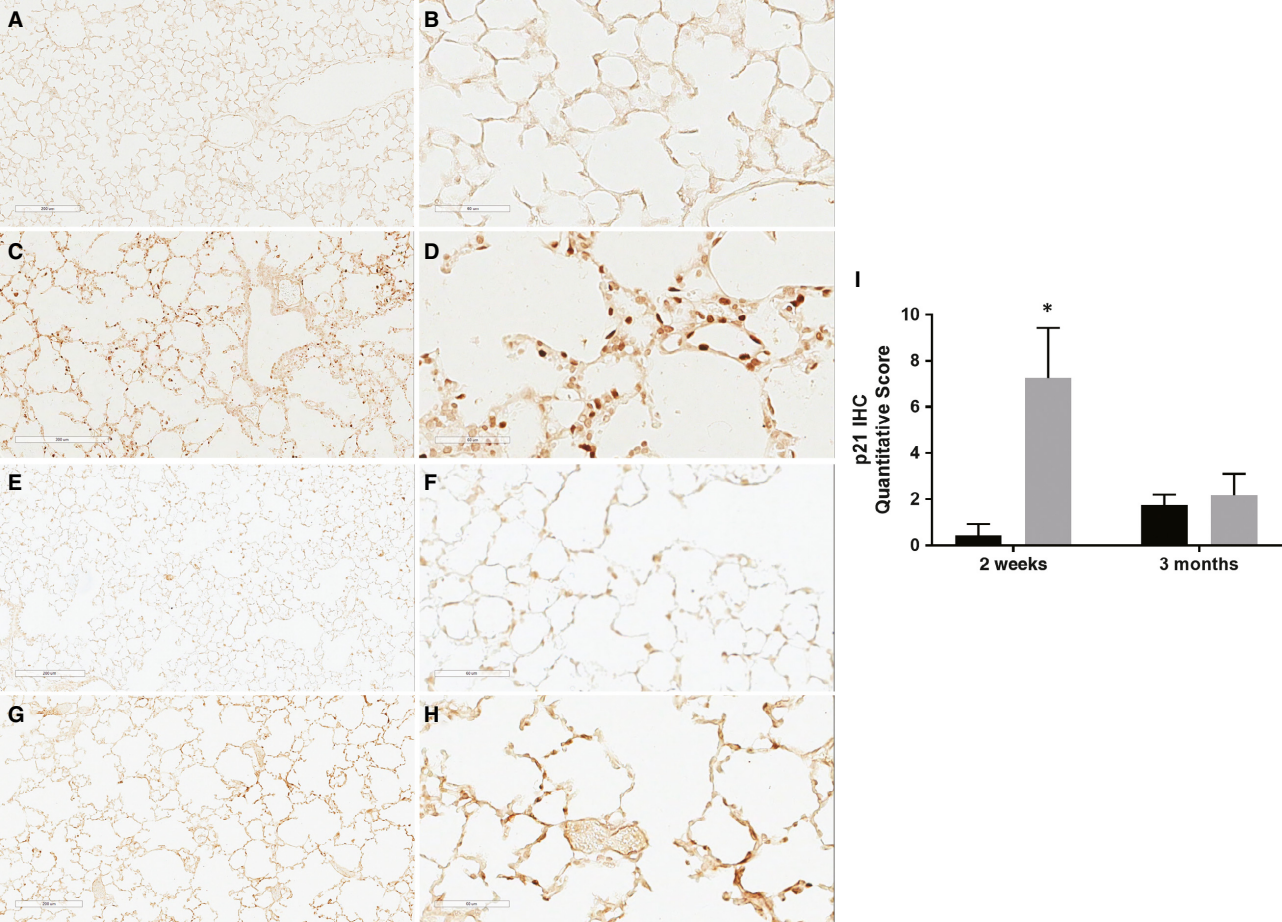
Immunohistochemistry for cell cycle inhibitor, p21, was performed in the lung at 2 weeks (A–D) and 3 months (E–H) in both the room air (A/B; E/F) and the hyperoxia (C/D; G/H) groups (100× – A, C, E, F; 400× – B, D, F, H). The staining quantified by intensity and the extent of staining (see text for details). Columns with black fill – room air groups; columns with gray fill – hyperoxia groups; *P < 0.05 versus room air – 2‐week group.

Nuclear staining for p21 protein was markedly intense and numerous in the 85% O_2_ group (Fig. 2C and D) with minimal to no staining in the room air group (2A/2B) at 2 weeks. On quantification of p21 immunohistochemistry, the scores were significantly higher in the hyperoxia group at 2 weeks (*P < 0.05 vs. room air group, Fig. 2I). Staining for p21 was present at 3 months; however, it was minimal to moderate, and there was no significant difference in the quantitative score between the two groups (2E/2F – room air, 2G/2H – hyperoxia, Fig. 2I).

## Discussion

Hyperoxia during the saccular stages of development in mice for 4–10 days induce alveolar simplification and structural and functional alterations in the lung (Veness‐Meehan et al. 2000, 2002; O'Reilly et al. 2008; Kumar et al. 2016) very similar to changes in infants with bronchopulmonary dysplasia. Infants with BPD are at increased risk for respiratory infections, airway reactivity, and alterations in function of the lung even in late childhood and early adult years (Doyle et al. 2006; Greenough 2008; Fawke et al. 2010). Mice exposed to neonatal hyperoxia do poorly upon challenge with influenza virus as adults, with increased severity of infection, greater weight loss, recruitment of inflammatory cells, and poor survival (O'Reilly et al. 2008). Hence, we studied the hyperoxia model of lung injury to investigate the immune responses in adult mice. Our results suggests that perinatal oxygen exposure may alter the developmental dynamics of immune response and the cell cycle regulator, p21, may play a role in the alteration of this response following neonatal hyperoxia.

Genes studied by the PCR array was a broad representation of the expression of T‐cell and B‐cell activation, proliferation, and differentiation along with genes regulating Th1 and Th2 development, a key component of adaptive immunity. Neonatal hyperoxia for 12 days from Day 3 to Day 15 suppresses the expression of genes involved in adaptive immunity during the critical phase of lung development and immune maturation. More than 25% of the genes studied were downregulated by ≥4‐fold, compared with the room air group by 2 weeks. T‐cell receptor signaling pathway was significantly downregulated in infants with BPD through the first month of life (Pietrzyk et al. 2013). A critical component of innate immune response involves presentation of the lipid antigen by CD1d1 to NK T cells to produce cytokines and activate adaptive immune responses. Hyperoxia downregulates the expression of CD1d1, one of the key immunoregulatory molecule, a critical first step, in protection against viral immunity. Neonatal hyperoxia has been shown to alter NK responses to influenza A virus infection in adult mice (Reilly et al. 2015). Expression of CD28 molecule, which transduces a positive signal to promote proliferation of TCR (T‐cell receptor)‐stimulated T cells, was significantly downregulated by hyperoxia. CD28 prolong survival of activated T cells, induces memory phenotype and hence critical for viral immunity. Downregulation of recombinant activation gene‐1 (Rag‐1) contributes to the delay or dysfunctional maturation of B and T lymphocytes following hyperoxia exposure; the gene is essential for rearrangement of immunoglobulin and T‐cell molecules produced by lymphocytes.

The cyclin‐dependent kinase inhibitor p21, a checkpoint regulator of cell cycle, was significantly overexpressed following neonatal hyperoxia. p21, in addition to being a regulator of cell cycle, is involved in cell differentiation and DNA repair (Sheikh et al. 1997; Stein et al. 1999). Previous studies have shown that hyperoxia induces p21 in the neonatal lung (McGrath 1998; O'Reilly et al. 1998, 2001). Newborn p21 knockout (21^−/−^) mice exposed to hyperoxia had decreased survival and significantly larger alveoli than p21^−/−^ control lungs (McGrath‐Morrow et al. 2004); p21 may also be important during recovery from lung injury because it is associated with lower levels of oxidative stress contributing to significantly lesser abnormalities in the lung (McGrath‐Morrow et al. 2004). When cells are damaged, p21 is the predominant mediator of G_1_ checkpoint, protecting cells against genotoxic stress inducing growth arrest. Low levels of p21 inhibits cell proliferation and growth, whereas higher levels promote an anti‐apoptotic phenotype, allowing repair of damaged DNA that might otherwise be a stimulus for apoptosis (Vitiello et al. 2006). Inhibition of cell proliferation and growth by p21 can suppress the expression, differentiation, and maturation of adaptive immune responses during development. All immune response genes studied were downregulated following hyperoxia except for p21, which was overexpressed. Costimulatory molecule, CD28, promotes T‐cell viability via upregulation of the anti‐apoptotic molecule Bcl‐X_L_ (Jones et al. 2002). The p21 protein expression was significantly higher in the lung and by immunohistochemistry at 2 weeks. Higher levels of p21 protein might also promote an anti‐apoptotic effect by maintaining the expression of Bcl‐X_L_, allowing for repair of damaged DNA from hyperoxia (Vitiello et al. 2006).

The overexpression of immune genes by 3 months in the hyperoxia group is essentially a “catch‐up” in terms of expression of these genes. Despite a prolonged period of room air recovery, there were some differences in immune responses between the hyperoxia and room air groups at 3 months. Significant downregulation of interferon‐*γ*, interleukin‐27, and CD40 was noted in the hyperoxia group compared with the room air group at 3 months. Recovery from severe suppression of immune genes following hyperoxia may be prolonged and dysfunctional in adult mice. Downregulation of interferon‐*γ* may be related to higher p21 levels in the hyperoxia group at 3 months, as overexpressed p21 reduces T‐cell activation and interferon‐*γ* secretion (Daszkiewicz et al. 2015). CD40–CD40 ligand interactions are critical for development of CD4 T‐cell‐dependent effector functions. Lack of this important interaction results in greatly reduced activation of CD4 T cells (Grewal and Flavell 1996). Interleukin‐4 (IL‐4) and interleukin‐10 (IL‐10) protein was higher in the room air group at 3 months. In T cells, binding of IL‐4 to its receptors induces proliferation and differentiation into Th2 cells. Th1 cells are vital for cell‐mediated immunity, whereas Th2 provide help for B cells and promote class switching from IgM to IgG1 and IgE (Choi and Reiser 1998). Lower IL‐4 in the hyperoxia group may reflect either delayed maturation or dysfunctional B‐cell responses from neonatal hyperoxia. Interleukin‐10, an anti‐inflammatory molecule, has role in controlling inflammation, immunoregulation and in downregulation of expression of Th1 cytokines (Ouyang et al. 2011). Neonatal mouse pups exposed to hyperoxia dramatically reduced abundance of IL‐0 in the lung during postnatal lung maturation (Lignelli et al. 2017); however, IL‐10 applied by intraperitoneal injection in the mouse model of BPD was not able to influence stunted lung alveolarization (Lignelli et al. 2017). IL27 protein expression was lower following hyperoxia at 2 weeks. IL27 has the ability to directly modify CD4^+^ and CD8^+^ T‐cell effector functions to induce IL10 and can modulate the intensity and duration of T‐cell responses to inflammation and infection (Hunter and Kastelein 2012). Lower IL27 at 2 weeks seen in the hyperoxia group may permanently alter the flexibility in responding to infection, inflammation, and stress in adult mice.

The study was limited by the absence of allergen or viral challenge to study Th1 and Th2 immune responses in adult mice. Protein expression was limited to few immunoregulatory molecules. Our intention was to assess adaptive immune responses to hyperoxia, to select few immunoregulatory molecules for detail analysis. Delaying O_2_ administration until postnatal day 3 may have limited the applicability of the model to infants with BPD who experience injury in the early saccular stage of lung development. However, hyperoxia is capable of causing “arrest of alveolarization” when exposure occurs from P3 in rodents (Veness‐Meehan et al. 2000). Significant numbers of infants with minimal O_2_ requirements at birth need increasing O_2_ concentrations by 2–4 weeks of age (Charafeddine et al. 1999). Hyperoxic lung injury early in development can alter the regulation of immune gene expression with effects in adult mice. We have shown that immune response gene expression is developmentally regulated, with massive suppression of immune genes by hyperoxia. This lays the foundation for altered and dysfunctional adaptive immune response in adults. Despite recovery in room air for prolonged periods, the continued suppression of immune responses suggests the effects of neonatal hyperoxia. Key signaling molecules such as IL27, IL4, and IL10 and p21 may play significant role in altered immune responses in adults. Factors affecting the natural evolution of adaptive immune responses after birth are not known, especially in premature infants. Hyperoxia alter the developmental regulation of adaptive immune response over time. The role of signaling molecules as therapeutic targets not only to treat BPD but also to facilitate normal adaptive immune responses will have broad implications in the management of premature infants.

## Conflict of Interests

The authors have no conflicts of interests to declare.

## References

[phy213577-bib-0001] Allison, B. J. , K. J. Crossley , S. J. Flecknoe , P. G. Davis , C. J. Morley , R. Harding , et al. 2008 Ventilation of the very immature lung in utero induces injury and BPD‐like changes in lung structure in fetal sheep. Pediatr. Res. 64:387–392. 18552709 10.1203/PDR.0b013e318181e05e

[phy213577-bib-0002] Ambalavanan, N. , W. A. Carlo , C. T. D'Angio , S. A. McDonald , A. Das , D. Schendel , et al. 2009 Cytokines associated with bronchopulmonary dysplasia or death in extremely low birth weight infants. Pediatrics 123:1132–1141. 19336372 10.1542/peds.2008-0526 PMC2903210

[phy213577-bib-0003] Balany, J. , and V. Bhandari . 2015 Understanding the impact of infection, inflammation, and their persistence in the pathogenesis of bronchopulmonary dysplasia. Front. Med. (Lausanne). 2:90. 26734611 10.3389/fmed.2015.00090 PMC4685088

[phy213577-bib-0004] Bhandari, V. , and J. R. Gruen . 2006 The genetics of bronchopulmonary dysplasia. Semin. Perinatol. 30:185–191. 16860158 10.1053/j.semperi.2006.05.005

[phy213577-bib-0005] Charafeddine, L. , C. T. D'Angio , and D. L. Phelps . 1999 Atypical chronic lung disease patterns in neonates. Pediatrics 103(4 Pt 1):759–765. 10103299 10.1542/peds.103.4.759

[phy213577-bib-0006] Choi, P. , and H. Reiser . 1998 IL‐4: role in disease and regulation of production. Clin. Exp. Immunol. 113:317–319. 9737656 10.1046/j.1365-2249.1998.00690.x PMC1905061

[phy213577-bib-0007] Daszkiewicz, L. , C. Vazquez‐Mateo , G. Rackov , A. Ballesteros‐Tato , K. Weber , A. Madrigal‐Aviles , et al. 2015 Distinct p21 requirements for regulating normal and self‐reactive T cells through IFN‐gamma production. Sci. Rep. 5:7691. 25573673 10.1038/srep07691 PMC4287747

[phy213577-bib-0008] Doyle, L. W. , B. Faber , C. Callanan , N. Freezer , G. W. Ford , and N. M. Davis . 2006 Bronchopulmonary dysplasia in very low birth weight subjects and lung function in late adolescence. Pediatrics 118:108–113. 16818555 10.1542/peds.2005-2522

[phy213577-bib-0009] Eriksson, L. , B. Haglund , V. Odlind , M. Altman , and H. Kieler . 2014 Prenatal inflammatory risk factors for development of bronchopulmonary dysplasia. Pediatr. Pulmonol. 49:665–672. 24039136 10.1002/ppul.22881

[phy213577-bib-0010] Fawke, J. , S. Lum , J. Kirkby , E. Hennessy , N. Marlow , V. Rowell , et al. 2010 Lung function and respiratory symptoms at 11 years in children born extremely preterm: the EPICure study. Am. J. Respir. Crit. Care Med. 182:237–245. 20378729 10.1164/rccm.200912-1806OC PMC2913237

[phy213577-bib-0011] Gasparoni, A. , L. Ciardelli , A. Avanzini , A. M. Castellazzi , R. Carini , G. Rondini , et al. 2003 Age‐related changes in intracellular TH1/TH2 cytokine production, immunoproliferative T lymphocyte response and natural killer cell activity in newborns, children and adults. Biol. Neonate 84:297–303. 14593240 10.1159/000073638

[phy213577-bib-0012] Greenough, A. 2008 Long‐term pulmonary outcome in the preterm infant. Neonatology 93:324–327. 18525217 10.1159/000121459

[phy213577-bib-0013] Grewal, I. S. , and R. A. Flavell . 1996 The role of CD40 ligand in costimulation and T‐cell activation. Immunol. Rev. 153:85–106. 9010720 10.1111/j.1600-065x.1996.tb00921.x

[phy213577-bib-0014] Han, C. P. , L. F. Kok , P. H. Wang , T. S. Wu , Y. S. Tyan , Y. W. Cheng , et al. 2009 Scoring of p16(INK4a) immunohistochemistry based on independent nuclear staining alone can sufficiently distinguish between endocervical and endometrial adenocarcinomas in a tissue microarray study. Mod. Pathol. 22:797–806. 19347018 10.1038/modpathol.2009.31

[phy213577-bib-0015] Hoebe, K. , E. Janssen , and B. Beutler . 2004 The interface between innate and adaptive immunity. Nat. Immunol. 5:971–974. 15454919 10.1038/ni1004-971

[phy213577-bib-0016] Hunter, C. A. , and R. Kastelein . 2012 Interleukin‐27: balancing protective and pathological immunity. Immunity 37:960–969. 23244718 10.1016/j.immuni.2012.11.003 PMC3531794

[phy213577-bib-0017] Jobe, A. H. 2011 The new bronchopulmonary dysplasia. Curr. Opin. Pediatr. 23:167–172. 21169836 10.1097/MOP.0b013e3283423e6b PMC3265791

[phy213577-bib-0018] Jobe, A. H. , and S. G. Kallapur . 2010 Long term consequences of oxygen therapy in the neonatal period. Semin. Fetal Neonatal. Med. 15:230–235. 20452844 10.1016/j.siny.2010.03.007 PMC2910185

[phy213577-bib-0019] Jones, R. G. , A. R. Elford , M. J. Parsons , L. Wu , C. M. Krawczyk , W. C. Yeh , et al. 2002 CD28‐dependent activation of protein kinase B/Akt blocks Fas‐mediated apoptosis by preventing death‐inducing signaling complex assembly. J. Exp. Med. 196:335–348. 12163562 10.1084/jem.20020307 PMC2193932

[phy213577-bib-0020] Kumar, V. H. , S. Lakshminrusimha , S. Kishkurno , B. S. Paturi , S. F. Gugino , L. Nielsen , et al. 2016 Neonatal hyperoxia increases airway reactivity and inflammation in adult mice. Pediatr. Pulmonol. 51:1131–1141. 27116319 10.1002/ppul.23430

[phy213577-bib-0021] Lignelli, E. , W. Seeger , R. Morty . 2017 Interleukin‐10 in experimental bronchopulmonary dysplasia. Am. J. Respir. Crit. Care Med. 195:A6397.

[phy213577-bib-0022] McGrath, S. A. 1998 Induction of p21WAF/CIP1 during hyperoxia. Am. J. Respir. Cell Mol. Biol. 18:179–187. 9476904 10.1165/ajrcmb.18.2.2964m

[phy213577-bib-0023] McGrath‐Morrow, S. A. , C. Cho , S. Soutiere , W. Mitzner , and R. Tuder . 2004 The effect of neonatal hyperoxia on the lung of p21Waf1/Cip1/Sdi1‐deficient mice. Am. J. Respir. Cell Mol. Biol. 30:635–640. 14607813 10.1165/rcmb.2003-0049OC

[phy213577-bib-0024] Morrow, L. A. , B. D. Wagner , D. A. Ingram , B. B. Poindexter , K. Schibler , C. M. Cotten , et al. 2017 Antenatal determinants of bronchopulmonary dysplasia and late respiratory disease in preterm infants. Am. J. Respir. Crit. Care Med. 196:364–374. 28249118 10.1164/rccm.201612-2414OC PMC5549867

[phy213577-bib-0025] O'Reilly, M. A. , R. J. Staversky , R. H. Watkins , and W. M. Maniscalco . 1998 Accumulation of p21(Cip1/WAF1) during hyperoxic lung injury in mice. Am. J. Respir. Cell Mol. Biol. 19:777–785. 9806742 10.1165/ajrcmb.19.5.3200

[phy213577-bib-0026] O'Reilly, M. A. , R. J. Staversky , R. H. Watkins , C. K. Reed , K. L. de Mesy Jensen , J. N. Finkelstein , et al. 2001 The cyclin‐dependent kinase inhibitor p21 protects the lung from oxidative stress. Am. J. Respir. Cell Mol. Biol. 24:703–710. 11415935 10.1165/ajrcmb.24.6.4355

[phy213577-bib-0027] O'Reilly, M. A. , S. H. Marr , M. Yee , S. A. McGrath‐Morrow , and B. P. Lawrence . 2008 Neonatal hyperoxia enhances the inflammatory response in adult mice infected with influenza A virus. Am. J. Respir. Crit. Care Med. 177:1103–1110. 18292469 10.1164/rccm.200712-1839OC PMC2383992

[phy213577-bib-0028] Ouyang, W. , S. Rutz , N. K. Crellin , P. A. Valdez , and S. G. Hymowitz . 2011 Regulation and functions of the IL‐10 family of cytokines in inflammation and disease. Annu. Rev. Immunol. 29:71–109. 21166540 10.1146/annurev-immunol-031210-101312

[phy213577-bib-0029] Pietrzyk, J. J. , P. Kwinta , E. J. Wollen , M. Bik‐Multanowski , A. Madetko‐Talowska , C. C. Gunther , et al. 2013 Gene expression profiling in preterm infants: new aspects of bronchopulmonary dysplasia development. PLoS ONE 8:e78585. 24194948 10.1371/journal.pone.0078585 PMC3806835

[phy213577-bib-0030] Reilly, E. C. , K. C. Martin , G. B. Jin , M. Yee , M. A. O'Reilly , and B. P. Lawrence . 2015 Neonatal hyperoxia leads to persistent alterations in NK responses to influenza A virus infection. Am. J. Physiol. Lung Cell. Mol. Physiol. 308:L76–L85. 25381024 10.1152/ajplung.00233.2014 PMC4281699

[phy213577-bib-0031] Schmidt, B. , R. S. Roberts , P. G. Davis , L. W. Doyle , E. V. Asztalos , G. Opie , et al. 2015 Prediction of late death or disability at age 5 years using a count of 3 neonatal morbidities in very low birth weight infants. J. Pediatri.. 167:982–6 e2. 10.1016/j.jpeds.2015.07.067 26318030

[phy213577-bib-0032] Shah, J. , A. L. Jefferies , E. W. Yoon , S. K. Lee , P. S. Shah , and Neonatal N. Canadian . 2015 Risk factors and outcomes of late‐onset bacterial sepsis in preterm neonates born at < 32 weeks' gestation. Am. J. Perinatol. 32:675–682. 25486288 10.1055/s-0034-1393936

[phy213577-bib-0033] Sheikh, M. S. , Y. Q. Chen , M. L. Smith , and A. J. Jr Fornace . 1997 Role of p21Waf1/Cip1/Sdi1 in cell death and DNA repair as studied using a tetracycline‐inducible system in p53‐deficient cells. Oncogene 14:1875–1882. 9150394 10.1038/sj.onc.1201004

[phy213577-bib-0034] Stein, G. H. , L. F. Drullinger , A. Soulard , and V. Dulic . 1999 Differential roles for cyclin‐dependent kinase inhibitors p21 and p16 in the mechanisms of senescence and differentiation in human fibroblasts. Mol. Cell. Biol. 19:2109–2117. 10022898 10.1128/mcb.19.3.2109 PMC84004

[phy213577-bib-0035] Veness‐Meehan, K. A. , F. G. Jr Bottone , and A. D. Stiles . 2000 Effects of retinoic acid on airspace development and lung collagen in hyperoxia‐exposed newborn rats. Pediatr. Res. 48:434–444. 11004232 10.1203/00006450-200010000-00004

[phy213577-bib-0036] Veness‐Meehan, K. A. , R. A. Pierce , B. M. Moats‐Staats , and A. D. Stiles . 2002 Retinoic acid attenuates O_2_‐induced inhibition of lung septation. Am. J. Physiol. Lung Cell. Mol. Physiol. 283:L971–L980. 12376350 10.1152/ajplung.00266.2001

[phy213577-bib-0037] Vitiello, P. F. , R. J. Staversky , S. C. Gehen , C. J. Johnston , J. N. Finkelstein , T. W. Wright , et al. 2006 p21Cip1 protection against hyperoxia requires Bcl‐XL and is uncoupled from its ability to suppress growth. Am. J. Pathol. 168:1838–1847. 16723699 10.2353/ajpath.2006.051162 PMC1606637

